# Role of the PLASMIC Score in the Management of Thrombotic Thrombocytopenic Purpura

**DOI:** 10.7759/cureus.36188

**Published:** 2023-03-15

**Authors:** Abhinav Vyas, Sangeetha Isaac, Dania Kaur, Udit Yadav

**Affiliations:** 1 Department of Internal Medicine, North Alabama Medical Center, Florence, USA; 2 Department of Hematology, Mayo Clinic, Rochester, USA

**Keywords:** hemolytic anemia, thrombotic coagulopathy, therapeutic plasma exchange (tpe), plasmic, acquired ttp, plasmic score

## Abstract

We report the case of a middle-aged male presenting with fatigue and abdominal pain. Prompt investigations demonstrated microangiopathic hemolytic anemia and thrombocytopenia on a peripheral blood smear. Thrombotic thrombocytopenic purpura was suspected based on the PLASMIC score. The patient significantly improved with therapeutic plasma exchange and prednisone within the next few days. The disintegrin and metalloprotease with a thrombospondin type 1 motif, member 13 levels reduction is a definitive hallmark leading to microvascular thrombosis. However, some medical centers in the United States do not promptly have quick allowance to the levels. Hence, the PLASMIC score becomes imminent in initiating immediate management and preventing life-threatening complications.

## Introduction

Thrombotic thrombocytopenic purpura (TTP) is a rare hematologic disorder traditionally presenting with features that include thrombocytopenia, microangiopathic hemolytic anemia, fever, neurological abnormalities, and acute kidney injury. The classical hallmark is a severe disintegrin and Metalloprotease with a thrombospondin type 1 motif, member 13 (ADAMTS-13) protease deficiency that leads to accumulation of ultra-large von Willebrand factor, subsequently causing microvascular thrombosis and hemolytic anemia. The syndrome is characterized by fever, thrombocytopenia, hemolytic anemia, neurologic symptoms, and renal abnormalities [1-3].

Severe ADAMTS-13 deficiency is diagnostic of TTP. However, resources to test for ADAMTS-13 levels may not be available at all centers. The PLASMIC score can predict ADAMTS-13 deficiency in suspected TTP with a high discrimination index. Here, we present the case of a patient diagnosed with TTP and successfully managed, in the absence of reliable ADAMTS-13 levels, solely based on the PLASMIC score. We also talk about how the PLASMIC score can be beneficial in differentiating between TTP and TTP mimics such as pseudo-thrombotic microangiopathy.

## Case presentation

A 44-year-old African American gentleman presented to the emergency department with complaints of abdominal pain and for the evaluation of low platelet counts and hemoglobin. In the background, the patient had been having left upper quadrant abdominal pain and dark stools for one month. The pain was burning in nature, and he graded it 10/10 in intensity. Further associated symptoms included shortness of breath, generalized weakness, fatigue, and occasional low-grade fevers. In addition, he reported a loss of weight of 16 pounds in the past two months. There was no history of recent abdominal surgery, blood transfusion, or musculoskeletal pain.

His past medical history was significant for peripheral artery disease status post-bilateral stenting of left and right common iliac arteries, cardiovascular hypertension, hyperlipidemia, and gastroesophageal reflux disease. Family history was significant for sickle cell anemia in two brothers and sickle cell trait mother and father. Of note, he did not have sickle cell disease. He was never diagnosed with anemia or any hematologic disorder in the past.

Initial examination in the emergency department demonstrated a pulse of 116 beats per minute, respiratory rate of 16 breaths per minute, and blood pressure of 145/83 mmHg, saturating 96% on room air. He was well-developed, well-nourished, and alert on physical examination, with mild acute distress secondary to abdominal pain. He was noted to have pale conjunctiva and mild conjunctival icterus. The cardiopulmonary examination was unremarkable. Abdominal examination revealed left upper quadrant tenderness; no bruit or organomegaly could be appreciated. Neurological examination demonstrated no motor-sensory deficits and was essentially unremarkable. Initial investigations demonstrated hemoglobin of 6.9 g/dL (13.8-17.2 g/dL), hematocrit of 19.8 L, mean corpuscular volume (MCV) of 88.7 fL, and platelet of 17,000 per µL (150,000-450,000 per µL) on the complete blood count panel. In addition, the comprehensive metabolic profile demonstrated elevated indirect bilirubin 2.0 mg/dL, aspartate aminotransferase (AST) 127 U/L (8-33 U/L), and alanine transaminase (ALT) 27 U/L (7-55 U/L). Other metabolic panel levels were within normal limits, including blood urea nitrogen (20 mg/dL) and serum creatinine (1.0 mg/dL). Initial labs are presented in Table 1.

**Table 1:** Lab values of our patient on presentation. MCV: mean corpuscular volume; RDW: red cell distribution width; BUN: blood urea nitrogen; LDH: lactate dehydrogenase; INR: international normalized ratio

Lab values	Reported value	Reference value range
Hemoglobin	6.9 g/dL	14.0–18.0
Hematocrit	19.8%	40.0–54.0
MCV	88.7 fL	80.0–94.0
RDW	19.8%	8.0–14.5
Platelet count	17 × 10E3/µL	150–375
BUN	20 mg/dL	4–22
Creatinine	1.0 mg/dL	0.6–1.3
Total bilirubin	2.2 mg/dL	0–1.0
Direct bilirubin	0.2 mg/dL	0–0.9
LDH	2298 U/L	313–618
Haptoglobin	<20 mg/dL	30–200
Vitamin B12	338 pg/mL	243–894
Reticulocyte count	13.2%	0.5–2.0
INR	1.0	0.8–1.2
Prothrombin time	10.8 seconds	9.0–11.6
Homocysteine	19.1 µmol/L	0.0–14.5

**Figure 1:**
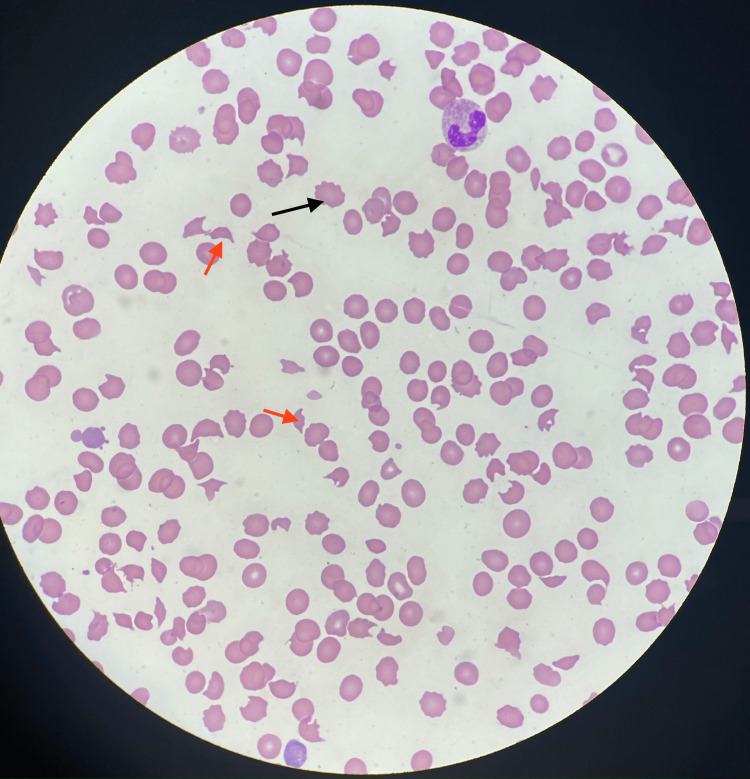
Microangiopathic process with schistocytes and acanthocytes. Red solid arrows: schistocytes/red blood cell fragments; black solid arrows: acanthocytes.

A hemolysis workup revealed low haptoglobin of fewer than 20 mg/dL (41-165 mg/L). Lactate dehydrogenase (LDH) was elevated at 2,298 U/L (105-333 U/L), fibrinogen was low at 85 mg/dL (200-400 mg/dL), and D-dimer was elevated at 3.88 mg/L (0-0.50 mg/dL). The coagulation profile was within normal limits, including prothrombin time (PT) and activated partial thromboplastin time (aPTT). Reticulocyte count was elevated at 13.2% (0.5-2.5%). Homocysteine was elevated at 19.5 µmol/L (5-15 µmol/L). Vitamin B12 levels were normal, and folate was low. Computed tomography (CT) scan with contrast of the chest/abdomen and pelvis was unremarkable. Peripheral blood smear demonstrated moderate to marked normocytic normochromic anemia with marked thrombocytopenia, as well as histiocytosis with a microangiopathic process with schistocytes and acanthocytes (Figure 1).

The investigations were indicative of hemolysis. The PLASMIC score was calculated and noted to be 7, indicating a high risk for TTP. Therefore, ADAMTS-13 levels were requested, and the patient was initiated on plasma exchange emergently. The initial ADAMTS13 specimen was sent out to another facility and was later reported to be unsuitable. Repeat testing revealed a level of 66%; however, the sample was drawn after the initiation of plasma exchange, rendering the result uninterpretable. The patient was also started on prednisone 1 mg/kg daily and rituximab 375 mg/m^2^ after potential risks, such as hepatitis B infection, were ruled out.

The patient significantly improved with therapeutic plasma exchange and prednisone within the next few days. He underwent five sessions of therapeutic plasma exchange with a significant platelet increase to 400,000 per µL within five days after the first session. The markers for hemolysis, such as haptoglobin, LDH, and reticulocyte count, started to normalize in three to four days. We subsequently discharged him in acceptable condition with a hematology follow-up. The patient was then scheduled for a rituximab infusion IV 375 mg/m^2^ weekly as an outpatient after discharge. He completed four doses of rituximab at the hematology follow-up clinic. Unfortunately, the patient developed steroid-induced hyperglycemia in the hospital, which we managed with metformin therapy. Prednisone was tapered gradually in the next two months, and metformin was discontinued once his fasting blood glucose normalized.

## Discussion

A recently published clinical algorithm states that the PLASMIC score is an excellent diagnostic model for patients with severe ADAMTS-13 deficiency [1]. The PLASMIC score for TTP prediction comprises components such as platelet count, hemolysis, history of solid organ cancer or stem cell transplant, MCV less than 90, INR less than 1.5, and creatinine less than 2.0. A systematic review and meta-analysis from 2020 stated that a PLASMIC score of 5 or higher provided a sensitivity of 99% and specificity of 57% [3]. A score of 6 or higher lowers the sensitivity to 85% and increases the specificity to 89%. These results suggest that withholding therapy can be life-threatening with potentially fatal outcomes. All patients with a PLASMIC score of 5 or higher should be empirically treated for TTP unless there is an obvious alternative explanation for the individual’s clinical presentation. Another study provided data stating that 67% of patients with idiopathic TTP may not be severely deficient in ADAMTS-13 levels triggered by stimuli causing an autoimmune reactivity to ADAMTS-13 protease leading to thrombotic platelet aggregation [4,5]. The PLASMIC score is mentioned in Table 2 [3].

**Table 2:** PLASMIC score INR: international normalized ratio; MCV: mean corpuscular volume Interpretation of scores: 0-4: Low risk for severe ADAMTS-13 deficiency. 5: Intermediate risk for severe ADAMTS-13 deficiency. 6-7: High risk for severe ADAMTS-13 deficiency.

Parameter	Points
Platelet count <30 × 10^9^/L	1
Hemolysis (reticulocyte count >2.5%, haptoglobin undetectable, or indirect bilirubin >2.0 mg/dL)	1
No active cancer	1
No history of solid-organ or stem-cell transplant	1
MCV <90 fL	1
INR <1.5	1
Creatinine <2.0 mg/dL	1

The classic pentad of fever, hemolytic anemia, thrombocytopenia, neurologic manifestation, and acute kidney injury is present only in about 5% of the patients with TTP and cannot be relied upon. Essential symptoms that could guide us to therapy should be gastrointestinal (69%), weakness (63%), and minor neurological findings (26%) such as headache and confusion [6]. Our patient had all these symptoms but no significant neurological symptoms or acute kidney injury. Therefore, in a clinical scenario with a high index of suspicion, the threshold to order hemolytic markers and calculate the PLASMIC score should be low.

A study by Li et al. concluded that patients with a high risk of TTP (PLASMIC score 6-7) had significantly more prolonged survival (p-value <0.01) with plasma exchange compared to low-to-intermediate-risk TTP (PLASMIC score 0-5) which had no difference in survival with the same intervention [1]. Before plasma therapy was found to be therapeutic, the mortality rate from TTP was as high as 90%. Hence, immediate management with therapeutic plasma exchange becomes essential, seeing the catastrophic course of the disease [7-9]. Our patient presented an unconventional presentation; however, with the help of diagnostic clues, we could immediately start the targeted therapy for TTP [10,11].

There have been a few reported cases of pseudo-thrombotic microangiopathy (TMA) that can be caused by vitamin B12 deficiency. The PLASMIC score considers MCV, which was less than 90 fL in our patient. Therefore, physicians should check vitamin B12 levels to rule out nutritional deficiencies. However, ADAMTS-13 levels in these patients are usually within normal limits in these subsets of patients [12]. Consequently, vitamin B12 replacement remains the treatment of such patients instead of therapeutic plasma exchange. Additionally, case reports have mentioned thiamine deficiency contributing to vitamin B12 deficiency causing pseudo-thrombotic microangiopathy [13].

Several case reports have attempted to develop a specific diagnostic criterion for pseudo-TMA with vitamin B12 deficiency. Some pertinent characteristics included hypersegmented neutrophils, low reticulocyte index, low to medium plasmic score, etc. However, our patient’s labs did not align with these diagnostic criteria. Hence, with the help of a peripheral blood smear, the PLASMIC score can help us differentiate between pseudo-TMA and TTP [13]. One retrospective cohort study in a premier institute in the United States showed higher PLASMIC scores in the TTP cohort compared to the pseudo-TMA cohort (6.1 points out of a possible 7 vs. 4.9, p-value = 0.02) [14].

## Conclusions

At centers that are resource-limited and where ADAMTS-13 testing is not readily available, it is imminent that treatment is started immediately based on the clinical presentation and PLASMIC score. A PLASMIC score can be relied on if or when ADAMTS-13 levels would take significant time before revealing the results. In this hematologic emergency, failure to initiate immediate measures, such as therapeutic plasma exchange and steroids, would prove detrimental to patients. In addition, a PLASMIC score is an excellent tool that can effectively differentiate between TTP and pseudo-TMA, such as vitamin B12 deficiency.

## References

[1] Li A, Khalighi PR, Wu Q, Garcia DA (2018). External validation of the PLASMIC score: a clinical prediction tool for thrombotic thrombocytopenic purpura diagnosis and treatment. J Thromb Haemost.

[2] Furlan M, Robles R, Galbusera M (1998). von Willebrand factor-cleaving protease in thrombotic thrombocytopenic purpura and the hemolytic-uremic syndrome. N Engl J Med.

[3] Paydary K, Banwell E, Tong J, Chen Y, Cuker A (2020). Diagnostic accuracy of the PLASMIC score in patients with suspected thrombotic thrombocytopenic purpura: a systematic review and meta-analysis. Transfusion.

[4] Tsai HM, Lian EC (1998). Antibodies to von Willebrand factor-cleaving protease in acute thrombotic thrombocytopenic purpura. N Engl J Med.

[5] Lian EC (2005). Pathogenesis of thrombotic thrombocytopenic purpura: ADAMTS13 deficiency and beyond. Semin Thromb Hemost.

[6] Bell WR, Braine HG, Ness PM, Kickler TS (1991). Improved survival in thrombotic thrombocytopenic purpura-hemolytic uremic syndrome. Clinical experience in 108 patients. N Engl J Med.

[7] Page EE, Kremer Hovinga JA, Terrell DR, Vesely SK, George JN (2017). Thrombotic thrombocytopenic purpura: diagnostic criteria, clinical features, and long-term outcomes from 1995 through 2015. Blood Adv.

[8] Singer K (1954). Thrombotic thrombocytopenic purpura. Adv Intern Med.

[9] Bernheim M, Roget J, Larbre F, Beaudoing A, Mouriquand C, Bethenod M, Jarlot B (1957). [Acute thrombotic thrombocytopenic purpura with hemolytic anemia; thrombotic microangiopathy; Moschcowitz disease]. Sem Hop.

[10] Mitra D, Jaffe EA, Weksler B, Hajjar KA, Soderland C, Laurence J (1997). Thrombotic thrombocytopenic purpura and sporadic hemolytic-uremic syndrome plasmas induce apoptosis in restricted lineages of human microvascular endothelial cells. Blood.

[11] Straat M, Müller MC, Meijers JC (2015). Effect of transfusion of fresh frozen plasma on parameters of endothelial condition and inflammatory status in non-bleeding critically ill patients: a prospective substudy of a randomized trial. Crit Care.

[12] Fahmawi Y, Campos Y, Khushman M (2019). Vitamin B12 deficiency presenting as pseudo-thrombotic microangiopathy: a case report and literature review. Clin Pharmacol.

[13] Ganipisetti VM, Bollimunta P, Tun NN, Kanugula A, Anil V, Athavale A, Maringanti BS (2023). Concomitant vitamin B1 and vitamin B12 deficiency mimicking thrombotic thrombocytopenic purpura. Cureus.

[14] Koshy AG, Freed JA (2020). Clinical features of vitamin B12 deficiency mimicking thrombotic microangiopathy. Br J Haematol.

